# Ibrutinib does not prevent kidney fibrosis following acute and chronic injury

**DOI:** 10.1038/s41598-021-91491-x

**Published:** 2021-06-07

**Authors:** Julie Belliere, Audrey Casemayou, Eloïse Colliou, Hélène El Hachem, Clément Kounde, Alexis Piedrafita, Guylène Feuillet, Joost P. Schanstra, Stanislas Faguer

**Affiliations:** 1 UMR 1297, Institut Des Maladies Métaboliques Et Cardiovasculaires, Institut National de La Santé Et de La Recherche Médicale (INSERM), Toulouse, France; 2 Université Paul Sabatier—Toulouse 3, Toulouse, France; 3 Département de Néphrologie Et Transplantation D’organes, Centre de Référence Des Maladies Rénales Rares, INSERM U1048 (I2MC, équipe 12), Centre Hospitalier Universitaire de Toulouse, 1, avenue du Pr Jean Poulhes, 31059 Toulouse, France

**Keywords:** Kidney, Inflammasome, Nephrology

## Abstract

Recent studies suggested that ibrutinib, a Bruton tyrosine kinase (BTK) inhibitor, developed for the treatment of chronic lymphocytic leukemia, may prevent NLRP3 inflammasome activation in macrophages, IL-1β secretion and subsequent development of inflammation and organ fibrosis. The role of NLRP3 has been underlined in the various causes of acute kidney injury (AKI), a pathology characterized by high morbimortality and risk of transition toward chronic kidney disease (CKD). We therefore hypothesized that the BTK-inhibitor ibrutinib could be a candidate drug for AKI treatment. Here, we observed in both an AKI model (glycerol-induced rhabdomyolysis) and a model of rapidly progressive kidney fibrosis (unilateral ureteral obstruction), that ibrutinib did not prevent inflammatory cell recruitment in the kidney and fibrosis. Moreover, ibrutinib pre-exposure led to high mortality rate owing to severer rhabdomyolysis and AKI. In vitro, ibrutinib potentiated or had no effect on the secretion of IL-1β by monocytes exposed to uromodulin or myoglobin, two danger-associated molecule patterns proteins involved in the AKI to CKD transition. According to these results, ibrutinib should not be considered a candidate drug for patients developing AKI.

## Introduction

Chronic kidney diseases (CKD) and acute kidney injury (AKI) are now recognized as diseases with global priority^1^. In 2017, more than 850 million individuals suffered from kidney disease^2^. To date, few treatments reduce the risk of CKD progression toward kidney failure, indicating the need for developing new therapeutic approaches. The clear link between AKI episodes and the development of CKD, or its progression toward kidney failure, has been recently emphasized owing to the findings of large epidemiological studies in human and experimental studies in animal models^3–6^. Preventing the AKI to CKD transition may reduce the burden of CKD worldwide and by extension progression to kidney failure. In line with this objective, repurposing of available and well-tolerated drugs according to their theoretical ability to target key molecular mechanisms of AKI and in the AKI to CKD transition may accelerate the transfer to the clinic.

AKI is a complex disease characterized by intra-renal hemodynamic changes and vascular hyperpermeability with subsequent heterogeneous tissue hypoxia, epithelial dysfunction and injuries, and sterile inflammation^7–9^. Renal inflammation is first characterized by the release of damage-associated molecular patterns (DAMPs) by injured epithelial cells and subsequent activation of macrophage Toll-like receptors (TLR), including TLR-4, in epithelial and immune cells. AKI is followed by successive waves of inflammatory cells infiltration, including monocytes, macrophages, neutrophils and ultimately B-cells^10^. It has been shown that targeting macrophages (early phase) or B-cells (late phase) can prevent AKI to CKD transition after ischemic or rhabdomyolysis-induced AKI^11,12^.

The binding of DAMPs to TLR-4 activates the NLR family, pyrin domain-containing 3 (NLRP3) inflammasome that mediates the release of the pro-inflammatory cytokines IL-1β and IL-18. The role of NLRP3 has been underlined in various causes of acute and chronic kidney diseases and NLRP3 inhibition or IL-1β blocking may protect from AKI and AKI to CKD transition^13^. Specifically, studies showed that DAMPs like uromodulin (formerly Tamm-Horsfall protein) crystals and probably myoglobin can activate NLRP3-dependent inflammation and participate to kidney injury^13,14^.

It has been suggested that the Bruton tyrosine kinase (BTK) binds to NLRP3 and can mediate its state of phosphorylation and therefore control NLRP3 activity^15,16^. Furthermore, these studies suggested that genetic or pharmacological inhibition of BTK can reduce the activation of NLRP3 in macrophages paving the way to interventional studies in NLRP3-dependent diseases using BTK inhibitors such as ibrutinib or acalabrutinib. BTK and NLRP3 being expressed in different cells involved in the pathogenesis of kidney injuries (i.e., epithelial cells, macrophages and B-cells), we put forward the hypothesis that ibrutinib may prevent AKI or transition from AKI to CKD by targeting immune and tubular cells. Unexpectedly, we observed that ibrutinib did not prevent fibrotic lesions after long-term follow-up in an *in-vivo* mouse model of AKI or in a rapidly progressive kidney fibrosis model. These data are in line with a very recent study suggesting that BTK inhibition actually sensitizes macrophages to TLR-4-induced NLRP3 activation and promotes injury-induced organ inflammation^17^ indicating to refrain from the use of ibrutinib, and probably BTK inhibitors in the treatment of AKI.

## Results

### Ibrutinib exacerbates UUO-induced renal fibrosis

**Figure 1:**
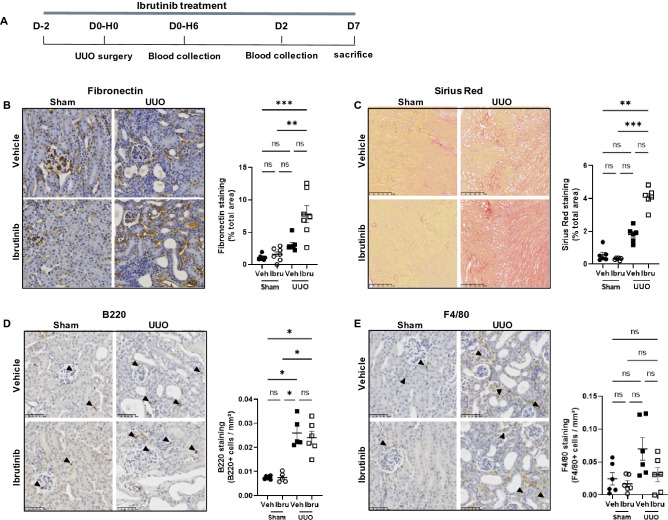
Ibrutinib promotes renal fibrosis after unilateral ureteric obstruction (UUO). (**A**) Design of the study. (**B**–**E**) Representatives images and quantifications of Fibronectin (**B**), Sirius Red (**C**), B220 (**D**) and F4/80 (**E**) staining in sham and UUO mice receiving ibrutinib (Ibru) or vehicle (Veh). Data are represented as means ± SEM. *p < 0.05 (Mann–Whitney test; n = 6 mice per group); ns, not significant.

We first tested the ability of ibrutinib to prevent renal injury and progression toward fibrosis in C57BL/6 mice submitted to unilateral ureteric obstruction (UUO), a model of rapidly progressive kidney fibrosis characterized by increased mRNA expression of *Il1b* and *Nlrp3*^18^. Daily ibrutinib treatment was started two days before UUO and pursued until sacrifice at day 7 (Fig. 1A). The mean serum level of ibrutinib was 11.5 ± 10 ng/ml at day 2. Seven days of UUO resulted in significant renal injury characterized by tubular dilatation, tubular cell swelling, tubular casts, necrotic cells within tubular lumen and interstitial edema (Fig. 1). UUO-induced renal fibrosis, quantified with fibronectin and red Sirius staining, was significantly higher in mice receiving ibrutinib (Fig. 1B,C). Furthermore, immunostaining of B-cell (B220) and macrophage (F4/80) markers was similar in ibrutinib and vehicle treated mice (Fig. 1D,E). mRNA expression of F4/80 was similar in both groups (data not shown). Thus, ibrutinib did not prevent renal fibrosis and did not reduce UUO-induced renal immune cell infiltration.

### Ibrutinib reduces rhabdomyolysis-induced survival and increases AKI severity

**Figure 2:**
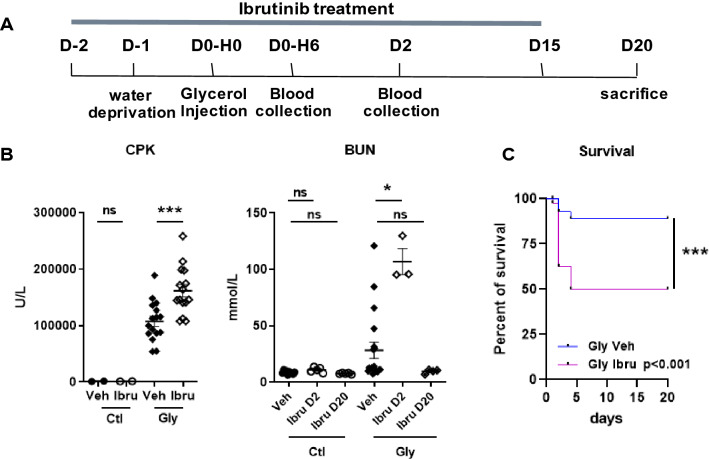
Prophylactic treatment with ibrutinib exacerbates rhabdomyolysis with subsequent severer acute kidney injury and higher mortality. (**A**) Design of the study. (**B**) Plasma level of CPK (6 h) and BUN (day 2 (D2) and 20 (D20)) after glycerol injection. (Mann–Whitney test and ANOVA, Tukey’s test; n = 3–15 mice per group). (**C**) Kaplan–Meier survival curves after glycerol injection in mice receiving ibrutinib (Ibru) or vehicle (Veh). Kaplan–Meier curves significance was calculated according to the Log-rank test. Values are expressed as mean ± SEM. *p < 0.05, ***p < 0.001, compared with the vehicle group; ns, not significant.

Since ibrutinib failed to prevent kidney injury and exacerbated the fibrotic phenotype in the UUO model, we verified the ability of ibrutinib to modify outcomes in mice submitted to rhabdomyolysis, a cause of AKI at risk of subsequent CKD also displaying increased expression of *Il1b* and *Nlrp3*^11^. C57BL/6 mice were exposed to glycerol-induced rhabdomyolysis after two days of ibrutinib administration. Ibrutinib was then pursued daily until day 15 (Fig. 2A). Compared to vehicle-treated animals, mice that received ibrutinib had higher CPK six hours after the glycerol injection (p < 0.001), higher BUN in surviving mice at day 2 (p < 0.05) (Fig. 2B) and higher mortality (p < 0.001) (Fig. 2C). Thus, mice receiving ibrutinib are sensitized to glycerol-induced rhabdomyolysis with subsequent severer AKI and higher mortality.

**Figure 3:**
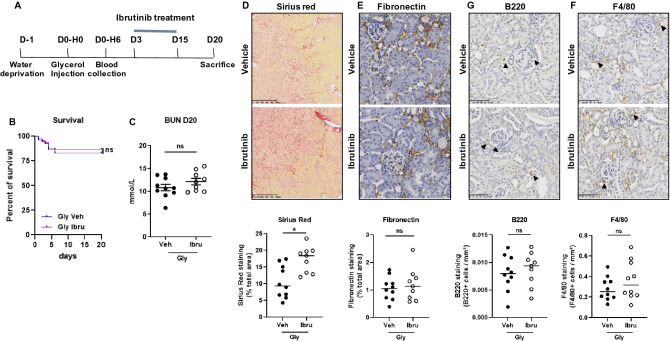
Ibrutinib promotes rhabdomyolysis-induced renal fibrosis. (**A**) Design of the study. (**B**) Kaplan–Meier survival curves after glycerol injection in mice receiving ibrutinib (Ibru) or vehicle (Veh) from day 3. (**C**) Blood urea nitrogen (BUN) 20 days after glycerol injection. (**D**–**G**) Representatives images and quantification of Sirius Red (**D**), Fibronectin (**E**), F4/80 (**F**) and B220 (**G**) staining 20 days after glycerol injection in mice receiving ibrutinib (Ibru) or vehicle (Veh) from day 3. Values are expressed as mean ± SEM. * p < 0.05, compared with the vehicle group (Mann–Whitney test, n = 5–10 mice); ns, not significant.

Because ibrutinib treatment led to a severer rhabdomyolysis and increased mortality when administrated before rhabdomyolysis, renal outcomes could not be adequately addressed. We therefore assessed the development of renal fibrosis following rhabdomyolysis in male mice receiving ibrutinib daily from day 3 to day 15 after glycerol injection (Fig. 3A). In this setting, survival of mice that received delayed ibrutinib was similar to control animals (Fig. 3B) and plasma BUN was similar in both groups at day 20 in those surviving animals (Fig. 3C). Red Sirius staining showed significantly more interstitial fibrosis in treated mice (Fig. 3D). Staining of fibronectin was similar in the two groups (Fig. 3E), as well as the mRNA expression of *Tgfb1* and *Tgfb2* (Supplementary Fig. 1). Again, the staining of the B-cell (B220) and macrophage (F4/80) markers was similar in ibrutinib and vehicle-treated mice (Fig. 3F,G). mRNA expression of F4/80 was increased in treated mice (Supplementary Fig. 1). Thus, ibrutinib treatment did not reduce rhabdomyolysis-induced renal immune cell infiltration nor renal fibrosis.

**Figure 4:**
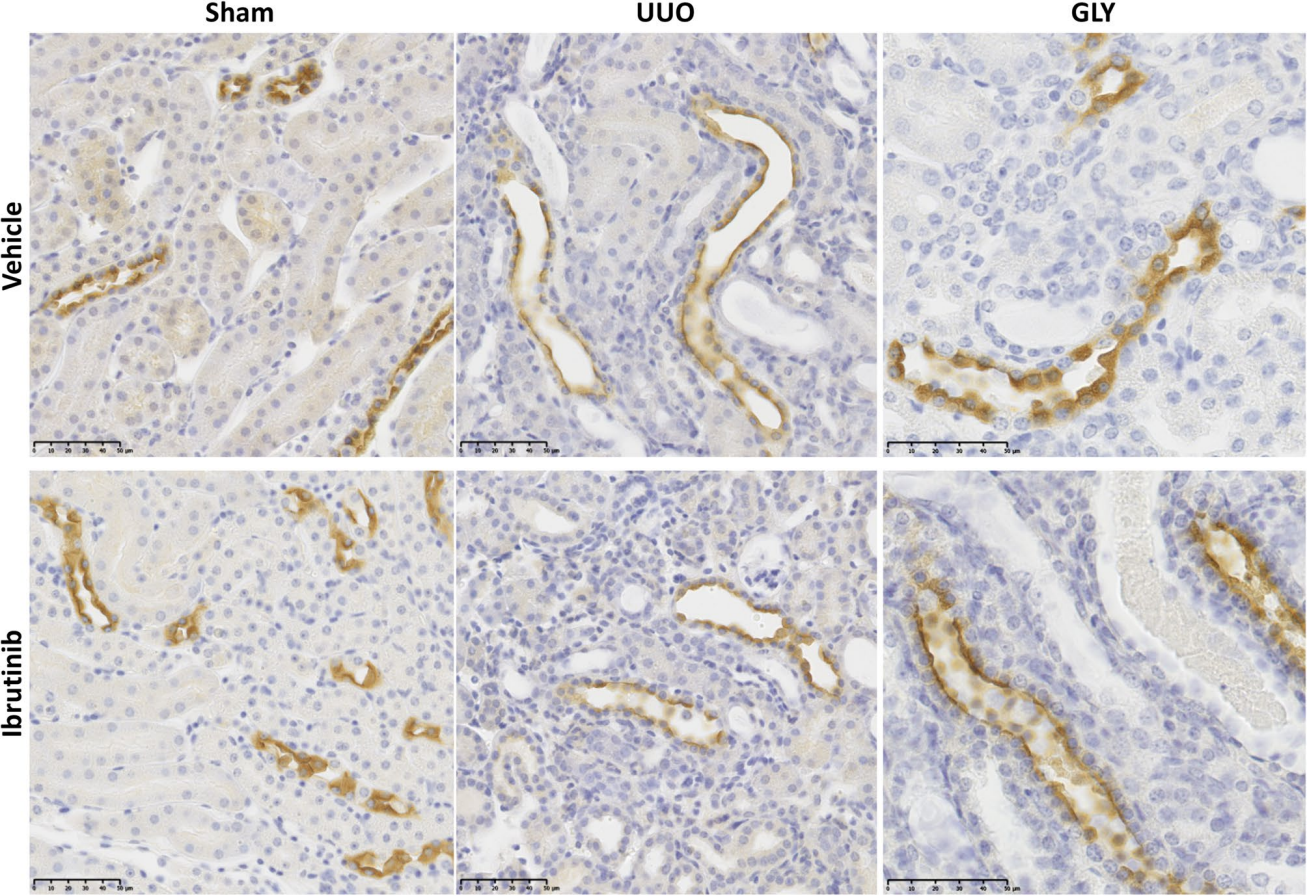
Kidney expression of BTK in sham and after glycerol-induced rhabdomyolysis (day 2; GLY) or unilateral ureteric obstruction (day 7, UUO).

To better understand this negative result, we characterized the expression of BTK in the kidney and showed that BTK is strongly expressed at the apical side of tubular cells within the medulla. Its expression was similar at baseline, after UUO and rhabdomyolysis, and in control and BTK-treated mice (Fig. 4). It was not identified in interstitial immune cells.

### Effects of ibrutinib treatment on DAMPs-induced cytokine production by monocytes/macrophages

**Figure 5:**
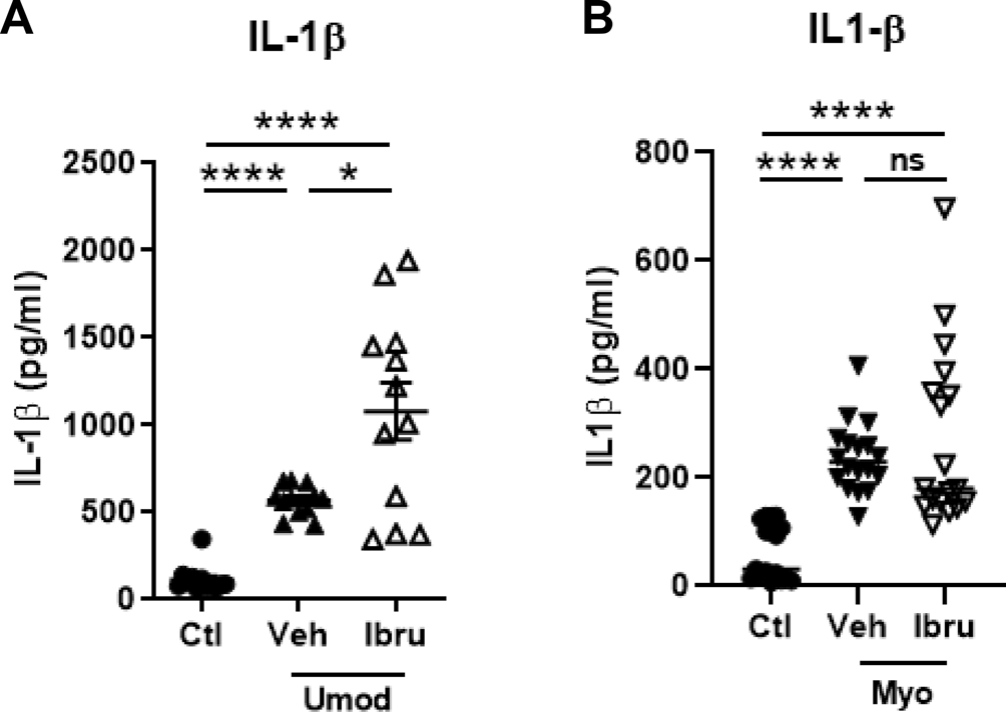
Effect of Ibrutinib on human PBMC treated with myoglobin or uromodulin. Concentration (pg/ml) of IL-1β assessed by ELISA in culture supernatant of human PBMC treated with Uromodulin (Umod, **A**) or Myoglobin (Myo, **B**) and with ibrutinib (Ibru) or vehicle (Veh). Data expressed as mean ± SEM, n = 4–12, *p < 0.05, ****p < 0.001 compared with the Veh or Ctl group (ANOVA Tukey’s test; n = 3–15 samples per group); ns, not significant.

Even if we did not find an effect of ibrutinib on macrophage number, to obtain further insight in the effects of BTK, we studied the effect of ibrutinib on macrophage activation following in vitro exposition to DAMPs. As a first step, human PBMC-derived monocytes were incubated for 24 h with uromodulin (Tamm-Horsfall protein), a potent TLR-4 and NLRP3 activator involved in mechanisms of AKI and transition toward CKD^14^. As shown in Fig. 5A, uromodulin induced the secretion of IL-1β, and exposure to ibrutinib increased the uromodulin-induced IL-1β excretion. As a second step, we reproduced these experiments by replacing uromodulin by myoglobin to mimic rhabdomyolysis. As shown in Fig. 5B, ibrutinib did not prevent myoglobin-induced IL-1β excretion. Myoglobin also increased the mRNA expression of *CCR7* and *TGFb1* but decreased the expression of *Dectin-1* (Supplementary Fig. 2). These *in-vitro* data using human cells confirmed the absence of protective effects of ibrutinib treatment and its potential harmful effects in cases of high TLR4-dependent NLRP3 activation with IL-1β over-secretion.

## Discussion

In the last years, CKD and associated cardiovascular complications has imposed a heavy burden on both patients and health budgets. Identifying new treatments to prevent progression of kidney disease toward end-stage kidney failure is mandatory but very few drugs successfully translated from animal models to humans. For example, targeting inflammatory processes that occur after AKI was often successful in mouse or rat models of transition from AKI to CKD, but these drugs are in most cases not available in humans or failed to show a benefit when evaluated in humans^11,19–21^.

Expression of the Bruton Tyrosine Kinase (BTK) in B-cells drives their B-Cell Receptor-dependent activation and survival^22^. BTK is also expressed in macrophages and some epithelial cells^23–25^ where it regulates the activity of the NLRP3 inflammasome, a key regulator of IL-1β secretion and innate immunity to sterile injuries. Ibrutinib inhibits BTK signaling in B-cells and revolutionized the prognosis of chronic lymphoid leukemia. Preventing B-cells activation with ibrutinib may also reverse autoimmunity in mouse and human, including lupus nephritis^26,27^. The role of B-cells in the development of renal fibrosis was demonstrated in three studies. One study showed that early-stage accumulation of B-cells after UUO led to extensive renal fibrosis, owing to increased mobilization and infiltration of monocytes/macrophages within scarred tissue^28^. In that study, CD20 antibody-mediated B-cell depleted mice had lower collagen deposition. This confirmed preliminary results obtained in a mouse model of ischemia/reperfusion renal injury where adoptive transfer of B-cells into B-cell deficient mice reduced tubular proliferation and increased tubular atrophy^12^. Very recently, the initial response to kidney injury following kidney transplantation in humans was linked with a late B-cell signature relating to renal dysfunction and fibrosis^29^.

Owing to these literature data, the role of NLRP3 in the development of AKI and progression toward CKD, and a preliminary study reporting beneficial effects of ibrutinib in cerebral ischemia-induced fibrosis, we hypothesized that BTK inhibition may reduce the severity of AKI and prevent the development of renal fibrosis. Unexpectedly, we observed in two different models of AKI that BTK inhibition does not prevent kidney injury. Increased renal fibrosis was suspected (increased red Sirius staining) but TGFb and collagens expressions were not modified. Whereas targeting B-cells may improve renal outcomes following AKI, we showed here that usual serum concentration of ibrutinib cannot prevent the trafficking of B220^+^ B-cells to the injured kidney following UUO and rhabdomyolysis induced-AKI^17^.

Secondly, we showed that ibrutinib instead of decreasing proinflammatory cytokine secretion either amplified DAMP-induced monocyte activation (uromodulin) or was without effect (myoglobin). Uromodulin and myoglobin are two DAMPs involved in kidney injury and/or promoting renal fibrosis^11,14,30^. Our data are in agreement with a recent study that reported that BTK deficiency sensitizes macrophages to NLRP3 inflammasome activation and subsequent IL-1β-dependent tissue injury^17^. In this study, authors showed that technical artifacts leading to incomplete TLR-4-induced NLRP3 activation led to misleading conclusions in previous studies that formerly reported NLRP3 down-regulation by ibrutinib^15,16^. Thus, our results obtained in vitro and in vivo strengthen these findings and suggest that ibrutinib may have detrimental effect in patients developing AKI with underlying strong TLR-4 and NLRP3 activation. A limitation of the use of ibrutinib is the fact that it does not selectively inhibit BTK, but can also inhibit the EGFR and other tyrosine kinases. Ibrutinib treatment thus can have potential BTK-independent effects. In addition, we showed that BTK is also expressed in epithelial tubular cells but its role in these cells remains to be established. Of note, ibrutinib had no effect on the kidney in control uninjured mice, supporting published randomized controlled trials that did not report spontaneous rhabdomyolysis or CKD in patients receiving ibrutinib. Therefore, adverse kidney events induced by ibrutinib may thus be only observed after AKI by mechanisms independent of immune cells.

Thirdly, we reported for the first time that mice receiving ibrutinib had severer rhabdomyolysis than controls. Our study was not designed to address the underlying mechanisms, but previous data showing macrophage infiltration and IL-1β secretion following muscle injury^31^ suggest that ibrutinib-induced increased activation of NLRP3 and subsequent secretion of IL-1β within muscles may have worsened the rhabdomyolysis. Whereas myalgia is a frequent adverse event in ibrutinib-treated patients, overt rhabdomyolysis has not been reported so far^32^, deserving further attention.

In summary, we show here that pharmacological inhibition of the Bruton Tyrosine Kinase does not prevent renal injury and instead increases renal fibrosis after UUO- and rhabdomyolysis suggesting that ibrutinib should not be considered a candidate drug for patients developing AKI or in transition from AKI to CKD. Our findings also suggest using ibrutinib with precaution in interventional studies testing its immunomodulation properties in refractory autoimmune diseases with kidney involvement, for instance in systemic lupus erythematosus.

## Methods

### Animals and procedures

#### Animals

C57BL/6 J mice (male, 8–10 weeks old) were purchased from Charles River and housed at a stable temperature and a 12-h light/dark cycle with free access to food and tap water. All reported experiments were conducted in accordance with National Institutes of Health guidelines for the care and use of laboratory animals and were approved by a local animal care and use committee (local approval number #122–2015-23). The study was carried out in compliance with the ARRIVE guidelines.

#### Experimental models

The UUO model was established as described previously^33^. Briefly, under oxygen-isoflurane anesthesia and through a longitudinal, left abdominal incision, the ureter was exposed and ligated with a 6/0 nylon thread at the uretero-pelvic junction. The contralateral kidneys were considered as sham. UUO mice were maintained on a standard mouse chow and tap water.

Before rhabdomyolysis induction, animals were deprived of water overnight. Rhabdomyolysis was induced in mildly sedated animals (isoflurane) by intramuscular injection in each thigh caudal muscle with 7.5 ml/kg 50% glycerol (VWR International, Radnor, Pennsylvania, USA) or saline as a control.

#### Treatment

BTK inhibitor ibrutinib (50 mg/kg/day, Euromedex, Souffelwersheim, France) or the same volume of vehicle (100 µl) was administered orally by gavage to the animals starting either two days before (preventive) or three days after (curative) glycerol injection. In the UUO model, ibrutinib was started 2 days before surgery.

#### Sample collection

Mice were sacrificed with a sublethal injection of Dolethal (0.182 mg per g of mice) and transcardially perfused with 2 ml PBS. Kidney were dissected and post-fixed in 4% PFA for 24 h at 4 °C for histology analysis.

Blood was collected from the mouse tail vein in EDTA tubes at 6 h, 2 or 20 days after glycerol injection or UUO and was centrifuged at 2000×*g* for 5 min to separate plasma. Plasma BUN and CPK was analyzed on a Pentra 400 analyzer (Horiba Medical, Grabels, France).

### Cell experiments

Peripheral blood mononuclear cells (PBMC) were isolated from the buffy coats of blood samples from healthy volunteers by centrifugation through a Ficoll-Paque PLUS density gradient (1,077 g/ml, GE Healthcare, Chicago, Illinois, USA). PBMC were washed thrice with PBS and 7.10^6^ cells were grown for 2 h in M-SFM medium (Thermo Fisher Scientific, Waltham, Massachusetts, USA). Monocytes were separated from PBMC by elimination of suspended cells. Then, PBMC-derived monocytes were cultured for 24 h with myoglobin (250 µM, M1882; Sigma-Aldrich, Saint-Quentin Fallavier, France), ibrutinib (3 µM, Imbruvica caps, Janssen Pharmaceutica, Belgium), uromodulin (6.25 mg/ml, Origen Biomedical, Burleson, Austin, USA), or medium alone.

Regarding the human blood samples, all the patients gave written informed consent and the samples collections was approved by ethical committee of the French Administration of Blood Products (Etablissement Français du Sang, authorization number 21PLER2017-0016). The study was performed according to the French law regarding human studies, and fulfilled the recommendations of the Helsinki’ conference, as revised in 2004.

### Pathology and Immunohistochemistry

Formalin fixed kidneys were embedded in paraffin, sectioned in 4 μm thick slices (whole cortex) and stained with filtered Sirius red 1% (BDH laboratories) or used for immunohistochemistry. For immunohistochemistry, anti-fibronectin, anti-B220 and anti-F4/80 primary antibodies (rabbit anti-fibronectin, 1/250^e^, Sigma-Aldrich, F3648; rat anti-B220, 1/500^e^, Ebioscience, 14–0452-81; rat anti-F4/80, 1/100^e^ Life technologies, MF48000, rabbit anti-BTK 1/500e Sigma #SAB3500372) were incubated for 1 h at room temperature. Following this, the specimens were washed twice with TBS 0,1% Tween 20 and incubated with Histofine simple stain MAX-PO (rabbit or rat, Nichirei, Tokyo, Japan) for 30 min and revelation was made with substrate Dako Envision system (K4010; Dako, les Ulis, France). Finally, sections were counterstained with hematoxylin then dehydrated and mounted. Negative controls for the immunohistochemical procedures included substitution of the primary antibody with non-immune sera. Sections were scanned using a Nanozoomer 2.0 RS (Hamamatsu Photonics SARL, Massy, France) and quantification of positive staining was performed using ImageJ.1 Fiji version software (https://imagej.net/downloads). Briefly, after deconvolution, positive staining is specified and pixels that satisfy the color specification are count by an algorithm in all over the scanned slide. Once the algorithm has been confirmed, the settings was saved in a macrofile for subsequent repeated use. Vessels staining were removed from the analysis.

### Statistical analyses

For statistical comparisons involving more than two experimental groups, one-way ANOVA was applied followed by Tukey post-hoc corrections. For comparisons between two data sets, Mann–Whitney tests were used. Survival curves were generated using the Kaplan-Maier approach and compared using the Log-rank test. A p-value below 0.05 was considered as significant. Statistical analyses were performed using the GraphPad software (Prism 8, San Diego, CA, USA).

## Supplementary Information

Supplementary Information.
