# Supplementary material for: Ibrutinib does not prevent kidney fibrosis following acute and chronic injury
Source: Sci Rep. 2021 Jun 7;11:11985. doi: 10.1038/s41598-021-91491-x (PMC8184891; doi:10.1038/s41598-021-91491-x)
Supplement: Supplementary file 1 — Supplementary Information. [file 41598_2021_91491_MOESM1_ESM.docx]

**Ibrutinib promotes kidney fibrosis following acute and chronic injury**

Julie BELLIERE^1,2,3,#^, Audrey CASEMAYOU^1,3,#^, Eloïse COLLIOU^1,2,3^, Hélène EL HACHEM^1,2,3^, Clément KOUNDE^1,2,3^, Alexis PIEDRAFITA^1,2,3^, Guylène FEUILLET^1,2^, Joost P. SCHANSTRA^1,2^, Stanislas FAGUER^1,2,3^

1 Institut National de la Santé et de la Recherche Médicale (INSERM), UMR 1297, Institut des Maladies Métaboliques et Cardiovasculaires, Toulouse, France

2 Université Paul Sabatier – Toulouse 3, Toulouse, France

3 Département de Néphrologie et Transplantation d’organes, Centre de référence des maladies rénales rares, Centre Hospitalier Universitaire de Toulouse, Toulouse, France

# These authors contributed equally to this work

**Supplementary Figure 1. mRNA expression of Tgfb1, Tgfb2 and F4/80 in kidneys of sham or after unilateral ureteric obstruction (UUO (A)) or glycerol-induced rhabdomyolysis (Gly (B)), and receiving or not ibrutinib.**

**
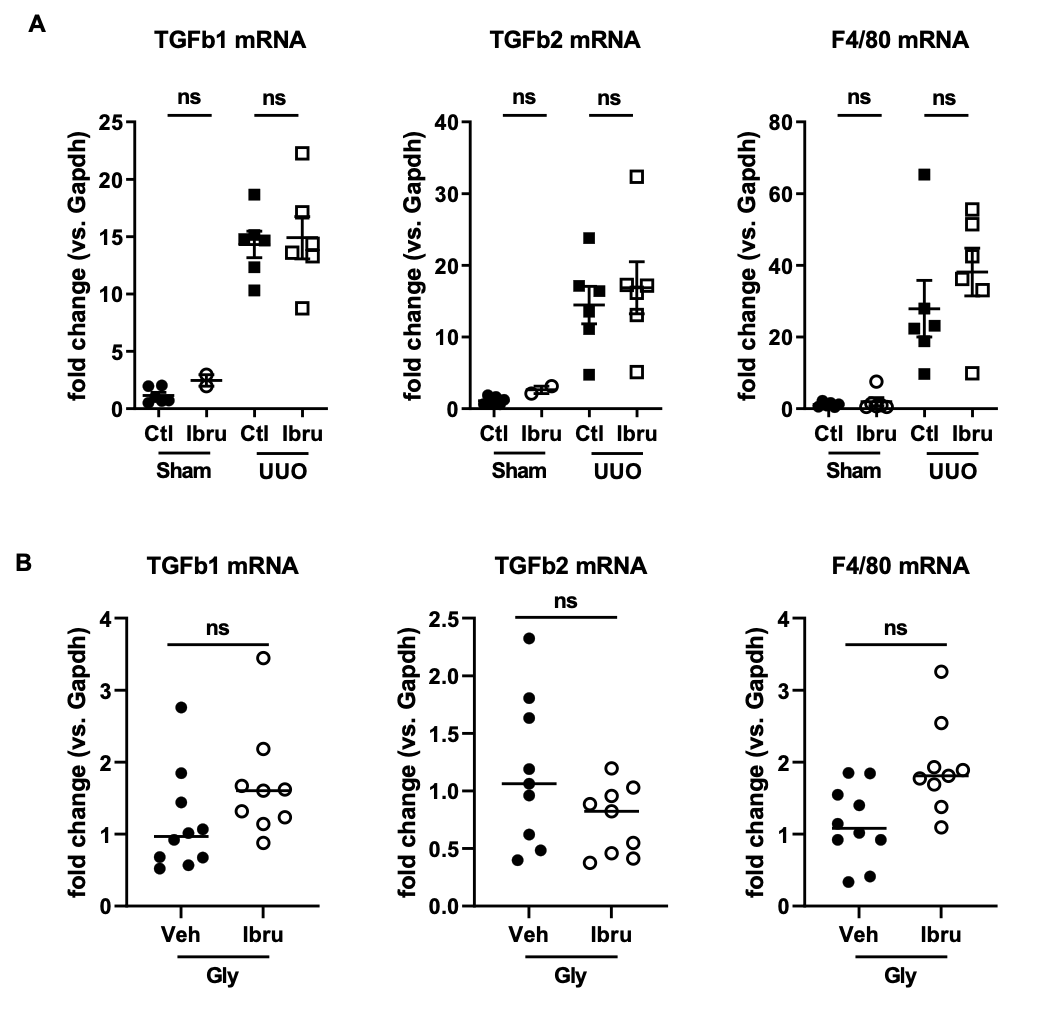
**

**Supplementary Figure 2. mRNA expression of *DECTIN1*, *CCR7* and *TGFB1* in peripheral blood mononuclear cells exposed to myoglobin ± ibrutinib.**

**
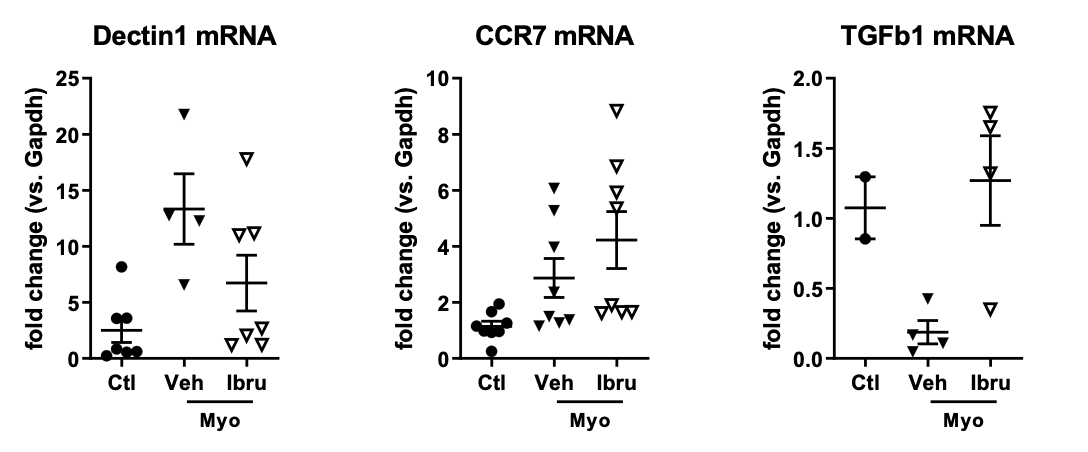
**
